# Genetic components of grey cattle in Estonia as revealed by microsatellite analysis using two Bayesian clustering methods

**DOI:** 10.1186/1756-0500-4-37

**Published:** 2011-02-11

**Authors:** Meng-Hua Li, Juha Kantanen, Annika Michelson, Urmas Saarma

**Affiliations:** 1 Biotechnology and Food Research, MTT Agrifood Research Finland, FI-31600 Jokioinen, Finland; 2 HAMK University of Applied Sciences, FI-31310 Mustiala, Finland; 3 Department of Zoology, Institute of Ecology and Earth Sciences, University of Tartu, 46 Vanemuise Street, EE-51014 Tartu, Estonia

## Abstract

**Background:**

It was recently postulated that a few individual grey cattle still found in Estonia might be a relict of the old native cattle stock. Genotypes at 17 microsatellite loci from a total of 243 cattle from North European breeds and 11 grey cattle in Estonia were used in an attempt to clarify the genetic composition of the grey cattle.

**Findings:**

We characterize the genetic components of 11 examples of the grey cattle in Estonia at the population and individual levels. Our results show that the grey cattle in Estonia are most genetically similar to the Holstein-Friesian breed and secondarily to the Estonian Red cattle.

**Conclusions:**

Both Bayesian approaches gave similar results in terms of the identification of numbers of clusters and the estimation of proportions of genetic components. This study suggested that the Estonian grey cattle included in the analysis are a genetic composite resulting from cross-breeding of European dairy breeds.

## Background

Conservation of farm animal genetic resources is of great value to the agricultural, economic, social and cultural sectors [1]. This is particularly true for native farm animals because the specific genes and gene combinations they carry may be useful, for example to cope with the challenge of global climate change (see [2]).

**Figure 1:**
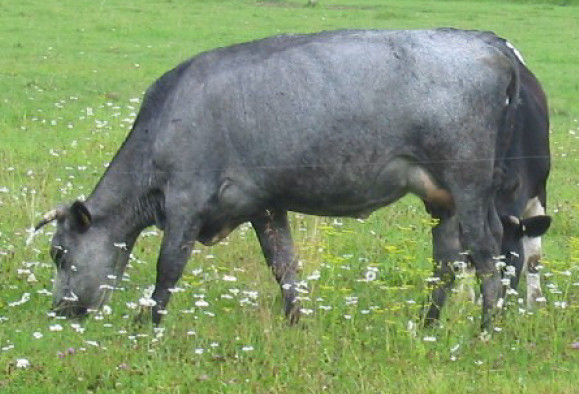
**A grey cow from the Vahtramäe farm in Estonia (Photo credit: Imbi Jäetma)**.

Baltic cattle populations have been greatly affected by a few productive breeds such as Danish Red, Angeln, and Holstein-Friesian [1]. As a result, only very few populations, e.g. the Estonian Native, are genetically characteristic of the native cattle that have survived in the Baltic countries [3]. Most of the original cattle have developed into new red- or black-pied breeds [1]. However, a recent survey suggested that there could exist local grey cattle in Estonia, with a total population size of *ca*. 60 animals, which were postulated to be a relict of the old native cattle stock [4,5]. Today, they are maintained in small herds owned by older farmers, and as such, there is limited pedigree information on these individuals. Typically, their hide is grey, blue grey, rot grey, ash grey, black and white (see Figure 1).

So far the genetic composition of grey cattle relative to other existing breeds in Estonia is still unknown. In this study we use a panel of 17 microsatellite loci and Bayesian-based assignment techniques to evaluate the relationship of Estonian grey cattle to other breeds occurring in North Europe.

## Methods

### Cattle samples and microsatellite data

Genotypes of a total of 254 animals from seven cattle populations (Grey cattle in Estonia, *n *= 11, see Table 1; Estonian Native, *n *= 40, Estonian Red, *n *= 40, Finnish Holstein-Friesian, *n *= 43, Latvian Blue, *n *= 40, Latvian Danish Red, *n *= 40, Latvian Brown, *n *= 40) were included in the analysis. Seventeen (*BM2113*, *HEL1*, *BM1824*, *BM1818*, *INRA032*, *INRA005*, *INRA035*, *ETH3*, *ILSTS006*, *HEL5*, *INRA023*, *INRA063*, *INRA037*, *ETH225*, *ILSTS005*, *CSSM66 *and *HEL13*) of 30 microsatellite loci recommended for genetic diversity studies in cattle http://www.projects.roslin.ac.uk/cdiv/markers.html were included in this investigation. The genotype data for the six parent populations were obtained from an earlier study [1].

**Table 1:** Data for the 11 grey cattle analysed in Estonia

Sample	County	Village	Gender	Colour
**Le1**	Läänemaa	Silla	♀	grey
**Le2**	Lääne-Virumaa	Kärsa	♀	grey
**Le3**	Lääne-Virumaa	Kärsa	♀	grey
**Le4**	Läänemaa	Kinki	♀	grey
**Le5**	Jõgevamaa	Maardla	♀	dark grey (blackish)
**Le6**	Jõgevamaa	Maardla	♀	grey (reddish)
**Le7**	Jõgevamaa	Maardla	♀	dark grey
**Le8**	Jõgevamaa	Maardla	♀	dark grey
**Le9**	Harjumaa	Rooküla	♂	grey
**Le10**	Harjumaa	Rooküla	♂	grey
**Le11**	Raplamaa	Laukna	♀	dark grey

Eleven Estonian grey cattle individuals from different stocks were blood-sampled. Particular efforts were made in all cases, using both the limited pedigree information (e.g. mostly only parent-offspring and full-sibling relationships) available and the knowledge of local herdsmen (e.g. the farm or village where the cattle originate from and the previous owners) via the interview questionnaire, to ensure that the animals were unrelated and had characteristics typical of the population [4]. Genomic DNA was extracted using a standard phenol/chloroform protocol [6]. PCRs were carried out following the protocols available at the Cattle Diversity Database http://www.projects.roslin.ac.uk/cdiv/markers.html. The size characterization of PCR products was done on a MegaBACE™ 500 capillary sequencer (GE Healthcare Life Sciences, Little Chalfont, UK) using the Fragment Profiler program ver. 1.2 (GE Healthcare Life Sciences). International control samples were also genotyped in order to standardize the size of allele fragments. Blood sampling of the 11 Grey cattle in Estonia was taken by a veterinarian in a procedure according to the Estonian Veterinary and Food Board and satisfied all ethical concerns.

### Data analysis

Tests for genotypic linkage disequilibrium (LD) for each locus pair and tests for deviation from Hardy-Weinberg equilibrium (HWE) were analysed in GENEPOP version 3.4 [7]. The global and pairwise genetic differentiation were determined as unbiased estimates of *F*_ST _[8] using FSTAT version 2.9.3.2 [9]. Significance of the results was established by applying sequential Bonferroni corrections (see [10]).

A Bayesian clustering method was first employed to assess population structure using the program STRUCTURE version 2.2 [11]. We performed 10 runs for each *K *value at 2 - 10 and ran the program assuming a model of admixture and correlated allele frequencies. We did not use any prior information about the population origin of the animals. A burn-in period of 200 000 generations and MCMC simulations of 500 000 iterations were used in all the above runs. The values of *Ln*P(D) (the log probability of data) were estimated assigning a prior from 2 to 10 and the optimal *K *was chosen based on the delta *K *(Δ*K*) value. This criterion was originally described in Evanno *et al. *[12] and was shown to be effective in later studies [1,13]. We then evaluated the population and individual membership coefficients (*Q*) of the 11 grey cattle in Estonia to the *K *inferred clusters.

BAPS version 5.4 [14] was run setting the maximum number of clusters at 20. Results were based on 50 simulations from the posterior allele frequencies. Since the mode of the posterior distribution of *K *almost always provided an overestimate of *K*, we used the number of clusters containing more than 3 individuals as a point estimate of *K*, as recommended by Tang *et al. *[14]. For runs in which *K *was correctly estimated, we calculated the average probability (*q*) of assignment to the 'correct' cluster ('correct' defined as *q *> 0.9 in the correct cluster). Individuals with a likelihood admixture ratio greater than 3.0 were considered to be significantly admixed.

## Results

The *F*_ST _analysis across breeds showed that 5.6% of the total genetic variation could be explained by the difference among populations. A low level of genetic differentiation was found between the grey cattle in Estonia and Finnish Holstein-Friesian (*F*_ST _= 5.2%; results not shown) as well as between the grey cattle in Estonia and the Estonia Red cattle (*F*_ST _= 5.6%; results not shown). Neither of the values was statistically significant at the 0.05 level (*P *> 0.05). No specific locus pairs showed a consistent deviation from LE that would have been in each, or even in most, of the populations. Deviations from HWE across the loci were present in the population of grey cattle in Estonia, which is most probably due to the small population size. However, no evidence for significant deviation from HWE was detected when a test was performed across all loci for all populations.

**Figure 2:**
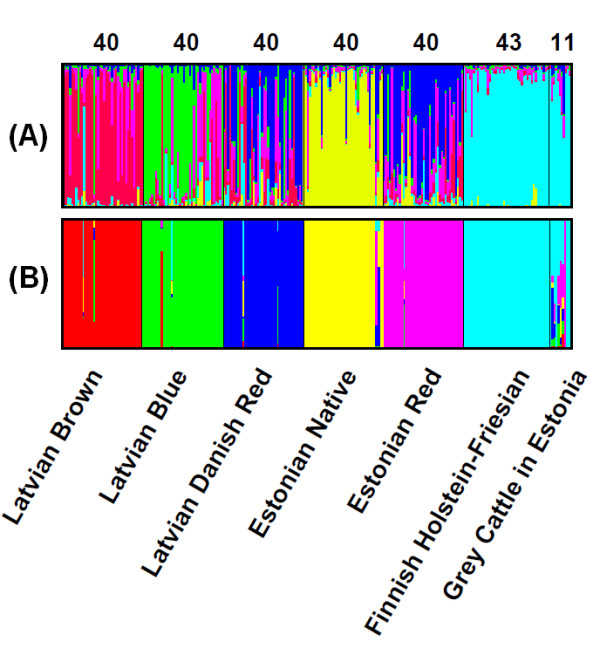
**Population structure of 7 cattle populations using**: **(A) **model-based STRUCTURE program (Pritchard *et al. *2000) and **(B) **BAPS program (Tang *et al. *2009). Each animal is represented by a single vertical line divided into *K *colours, where *K *is the number of clusters assumed. The coloured segment shows the individual's estimated proportion of membership (averaged across 10 runs at *K *= 6) in that cluster for the STRUCTURE program and indicates the average probability of assignment to the "correct" cluster for the BAPS program. Black lines separate the populations labelled above the figure. The labels above the figure indicate the number of animals analysed in each breed and the names of the cattle populations analysed are indicated below the figure.

Based on the population *Q*-values, the STRUCTURE program identified six clusters among the seven populations, but could not discern all seven populations (Figure 2A). More exactly, it failed to differentiate between the grey cattle in Estonia and Finnish Holstein-Friesian. Over the entire cattle populations, *Ln *P(D) increased from *K *= 2 to *K *= 6, after which it began to decline, indicating the most likely value to be *K *= 6 (results not shown). When we used ΔK to infer the number of clusters, we found that *K *= 6 was clearly favoured (results not shown). At *K *= 6, all the grey cattle in Estonia were characterized, with the highest proportion of membership from the Finnish Holstein-Friesian cluster (*Q*_FiHF_). Five grey cattle (Le1, Le4, Le9, Le10 and Le11) showed high values of *Q*_FiHF _> 0.9 and the remaining grey cattle are suggested to have large membership fractions in multiple clusters for the sampled populations. In particular, the grey cattle Le8 have similar values of *Q *for two distinct populations, Finnish Holstein-Friesian (*Q*_FiHF _= 0.480) and the Estonian Red (*Q*_EsR _= 0.351; Table 1).

With respect to the overall pattern of population clustering, results with BAPS were mostly consistent with those obtained with STRUCTURE. The analysis of population genetic structure carried out with BAPS suggested *K *= 6 to be the best clustering option (see Figure 2B), with the six clusters corresponding to the six source populations. Figure 2B shows the proportions of membership (*q*) of each grey cattle individual in each of the six identified clusters, while the corresponding values are presented in Table 2. Five samples (Le1, Le4, Le9, Le10 and Le11) exhibited *q *values of 100% for the cluster of Holstein-Friesian and one (Le8) for Estonian Red. The remaining samples received proportions of membership from multiple clusters, while the higher average proportion of their membership was from Finnish Holstein-Friesian followed by Estonian Red (Table 1).

**Table 2:** Membership proportions (*Q*) of the 11 grey cattle in Estonia for the 6 genetic clusters

Animals	Cluster 1 **(Latvian Brown)**^a^	Cluster 2 (Latvian Blue)	Cluster 3 (Latvian Danish Red)	Cluster 4 (Estonian Native)	Cluster 5 (Estonian Red)	Cluster 6 (Finnish Holstein-Friesian)
Le1	0.015(0.00)^b^	0.003(0.00)	0.006(0.00)	0.005(0.00)	0.008(0.00)	**0.963**(**1.00**)^c^
Le2	0.015(0.01)	0.015(0.04)	0.081(0.40)	0.015(0.03)	0.145(0.05)	0.729(0.47)
Le3	0.025(0.00)	0.017(0.06)	0.072(0.12)	0.008(0.00)	0.027(0.15)	0.852(0.67)
Le4	0.009(0.00)	0.019(0.00)	0.008(0.00)	0.003(0.00)	0.008(0.00)	**0.953**(**1.00**)
Le5	0.012(0.01)	0.023(0.15)	0.038(0.12)	0.016(0.02)	0.047(0.25)	0.864(0.45)
Le6	0.013(0.02)	0.008(0.00)	0.032(0.08)	0.029(0.25)	0.169(0.34)	0.749(0.31)
Le7	0.021(0.12)	0.036(0.10)	0.054(0.12)	0.009(0.10)	0.241(0.24)	0.639(0.32)
Le8	0.006(0.00)	0.007(0.00)	0.351(0.00)	0.009(0.00)	0.147(**1.00**)	0.480(0.00)
Le9	0.033(0.00)	0.009(0.00)	0.008(0.00)	0.012(0.00)	0.014(0.00)	**0.925**(**1.00**)
Le10	0.009(0.00)	0.005(0.00)	0.007(0.00)	0.010(0.00)	0.014(0.00)	**0.954**(**1.00**)
Le11	0.010(0.00)	0.035(0.00)	0.010(0.00)	0.014(0.00)	0.026(0.00)	**0.905**(**1.00**)

^a ^The genetic clusters inferred using the STRUCTURE program

^b ^The average probability (*q*, in the parentheses) of assignment to the "correct" cluster ("correct" defined as *q *> 0.9 in the correct cluster) by using the BAPS program

^c ^Values of *Q *> 0.9 or *q *> 0.9 are in bold

## Discussion

On-average we found higher proportions of membership for Finnish Holstein-Friesian and Estonian Red cattle in the grey cattle. The grey cattle represent a composite of North European cattle.

The composite genetic components may explain their distinctive grey colour, which is a mixture of colours. This finding is also evidenced by the fact that a grey cow sometimes has grey and/or black-and-white calves in the same birth. Although the grey cattle are characterized as having most of their genetic components from the black-and-white dairy cattle (*i.e. *Holstein-Friesian) or Estonian Red, they can be valuable in the investigation of the genetics of the colour genes.

Both STRUCTURE and BAPS correctly inferred the number of clusters in a dataset when genetic differentiation among populations was low. However, it seems that the proportions of individual membership in the clusters estimated by the program STRUCTURE are more consistent with the breeding history for the populations. For example, Latvian Danish Red, Estonian Red and Latvian Brown are the local derived populations from the Anglen and Danish Red cattle. This shared ancestry is reflected in the results of STRUCTURE, but not of BAPS. For the 11 grey cattle in Estonia, both programs gave comparable results for proportions of individual membership. To secure high confidence in results, we advocate using both programs for inferring the number of clusters and assignment of individuals to clusters, particularly when the level of genetic differentiation among populations is low.

Finally, a growing number of domestic animal populations are genotyped for the same panel of microsatellites (see [15]), for example the markers recommended by the FAO (Food and Agriculture Organization of the United Nations). This can help address similar kinds of questions on genetic components and the nature of native animal stocks because more data for potential reference and parental populations are available. The livestock populations for which there is a high priority for conservation, in terms of proportions of their native genetic components (e.g. [16-18]), can be identified and, thus, need to be included in conservation programmes in the near future.

## Conclusions

In conclusion, given the low levels of genetic differentiation among the populations investigated, both Bayesian approaches gave similar results in terms of identification of the numbers of clusters and the estimation of proportions of genetic components. Our study shows that the Estonian grey cattle analysed were a genetically admixed population, most influenced by the Holstein-Friesian and Estonian Red cattle.

## Competing interests

The authors declare that they have no competing interests.

## Authors' contributions

MHL designed the study, performed the data analysis and wrote the manuscript. JK planned and coordinated the whole study, and contributed to the manuscript writing. AM collected information about Estonian grey cattle, contacted cattle owners and interviewed them. US participated in study design, sample collection of grey cattle in Estonia and the preliminary data analysis. All the authors read and approved the final manuscript.
